# Information theoretical analysis of differences in information transmission in cerebellar Purkinje cells across species

**DOI:** 10.1186/1471-2202-15-S1-P40

**Published:** 2014-07-21

**Authors:** Kirsty Kidd, James M Bower, Daniel Polani, Neil Davey, Volker Steuber

**Affiliations:** 1 Science and Technology Research Institute, University of Hertfordshire, Hatfield, Hertfordshire, UK

## 

The dendrite of the cerebellar Purkinje cell is one of the most complex structures in the mammalian brain, receiving more than 150,000 synaptic inputs. It is also one of the most extensively modelled neurons in the mammalian brain, with theoretical analysis of the input-output relationships in its dendrite extending back 40 years. While most of this experimental and modelling work has been conducted using mammalian neurons, it has also often been noted that overall cerebellar structure as well as the general morphology of Purkinje cells has been highly conserved in all vertebrate species. The work described here seeks to identify conserved features of Purkinje cell function by examining the relationship between structure and function in a range of vertebrate species from fish to mammals.

This study is based on passive compartmental models constructed from anatomical reconstructions of Purkinje cells obtained from fish, reptiles, birds and mammals. Morphologically, while the Purkinje cells of each of the species are flattened structures and appear to have the same geometrical relationship to other elements of the cerebellar cortical circuitry, the dendrites of birds, alligators and mammals have a much more highly articulated dendritic branching structure than the dendrites of fish and turtles. In previous studies of the passive electrical properties of these dendrites [1], we have shown that there is surprisingly little difference in voltage attenuation between the species. In this extension of the comparative work, we have applied techniques from information theory to quantify possible similarities and differences in the effect of synaptic inputs on the passive properties of the dendrite. Specifically, we propose a new technique to find the time window that best predicts an output spike train given the input, and have used the weighted context tree method [2] to estimate the synaptic information efficacy (SIE) of individual synapses found in different locations on the dendritic tree.

## References

[B1] Kidd K Cornelis H Bower JM Polani D Davey N Steuber V The implications of evolutionary changes in the dendritic morphology of cerebellar Purkinje cells for information processing [abstract] BMC Neurosci 2013 15 Suppl 1 P373

[B2] London M Schreibman A Häusser M Larkum ME Segev I The information efficacy of a synapse Nat Neurosci 2002 15 4 332 340 10.1038/nn826 11896396

